# Toothbrush and toothpaste preferences among Southern Saudi children: insights into oral hygiene awareness and practices

**DOI:** 10.3389/fdmed.2026.1815634

**Published:** 2026-08-05

**Authors:** Zuhair Motlak Alkahtani, Hassan Ahmed Assiri, Haifa N. Alsharif, Ghydaa Sultan Al-Hufayyan, Jawharah Ali Y Mari, Rawan Saeed Shafloot, Nadiyah Abdullah Alasmari, Rahaf Alshehri, Sahar Alshamrani, Nada Yahya, Amit Porwal

**Affiliations:** 1 College of Dentistry, King Khalid University, Abha, Saudi Arabia; 2 Jazan University College of Dentistry, Jazan, Saudi Arabia

**Keywords:** children, oral hygiene, parental supervision, Saudi Arabia, toothbrush preference, toothpaste flavour

## Abstract

**Background:**

Oral health is an integral component of general well-being, and the development of good oral hygiene habits during childhood plays a vital role in preventing dental caries and periodontal diseases. Understanding children's preferences regarding toothbrushes and toothpastes can help design effective preventive strategies and enhance compliance with oral hygiene practices.

**Aim:**

To assess the awareness, behavior, and preferences of children regarding the selection and use of toothbrushes and toothpastes.

**Materials and methods:**

A cross-sectional study was conducted among 500 children aged 5–12 years who visited the Pediatric Dentistry Clinics of King Khalid University, College of Dentistry, from September 2023 to January 2024. A validated self-administered questionnaire with visual aids was used to record demographic data, brushing frequency, and preferences for toothbrush and toothpaste color, flavour, and design. Data were analyzed using IBM SPSS version 20.0, using descriptive statistics and the chi-square test (*p* < 0.05).

**Results:**

Most participants brushed their teeth twice daily (*p* < 0.05), with a majority brushing independently. Significant gender differences were found: females preferred pink toothpaste (29.2%)/brushes (38.0%), strawberry flavour (55.2%), and design 4 (44.0%), while males preferred blue toothpaste (38.4%)/brushes (45.6%), and mint flavour (32.0%). Males more often chose toothpaste independently (46.4% vs. 36.8%, *p* = 0.024) and a consistent brand (74.8% vs. 69.2%, *p* = 0.029).

**Conclusion:**

Children demonstrated satisfactory awareness and motivation toward maintaining oral hygiene. However, continued parental supervision and education remain essential to ensure correct brushing techniques and fluoride use. Incorporating sensory preferences, such as colour and flavour, into oral hygiene products may enhance children's compliance and foster lifelong healthy habits.

## Introduction

The oral cavity is often regarded as the mirror of general health, reflecting an individual's overall well-being. Oral and systemic health are closely interlinked, each influencing the other through complex interactions involving awareness, attitudes, and behaviours toward hygiene and disease prevention. Good oral health, defined as the absence of oral pain, infection, disease, or functional limitation, contributes directly to improved nutrition, communication, and psychosocial development (1). Conversely, poor oral health adversely affects quality of life and can impair speech, aesthetics, and social interaction. Because of the high global burden of oral diseases, oral health has now been recognized as a critical component of general health (2, 3).

Maintaining oral hygiene is a foundation of preventive dentistry. Two fundamental aspects determine oral hygiene: individual self-care practices, such as toothbrushing, dietary control, and fluoride application; and professional dental care, including routine checkups, health education, and preventive interventions (4–6) Although considerable advances have been made in preventive dentistry, the prevalence of common oral diseases such as dental caries and periodontal disease remains high across many populations (7). The high prevalence of oral conditions depends on the oral hygiene behavior, inadequate hygiene practice, and oral hygiene awareness programs in the population (8, 9).

It is well established that the foundation for lifelong oral health is laid in early childhood. Habits formed during this period are difficult to modify later in life. Therefore, promoting positive oral hygiene behaviours among children is essential. Parents, teachers, and dental professionals share a collective responsibility to instil these behaviours through guidance, modelling, and reinforcement (9–11). Preventive approaches remain the basis of modern dental practice, and the use of appropriate oral-cleaning aids, such as toothbrushes, toothpastes, dental floss, and mouthrinses, has long been recognized as fundamental to daily hygiene maintenance (2, 4).

Among these aids, toothbrushes and toothpastes play a particularly significant role in shaping children's interest and motivation toward toothbrushing. For young users, sensory and visual factors such as color, flavour, and packaging design strongly influence preference and compliance (12). Toothpaste packaging and appearance act as visual cues that attract attention and foster brand recognition (13). Children's limited cognitive capacity makes them highly responsive to such sensory attributes, often prioritizing visual appeal over functional benefits (13). As previous marketing-based studies suggest, color and flavor perception are closely linked to emotional engagement and taste expectations, influencing not only purchasing decisions but also the regularity of use (5, 6, 13, 14).

Color, in particular, has been identified as the most powerful intrinsic sensory cue shaping expectations of taste and satisfaction (12). Children tend to associate bright, vivid colors such as red or blue with sweetness or a pleasant flavor, whereas pale shades may evoke medicinal connotations. Similarly, fruity, minty, or sweet smell and flavor preferences can significantly enhance brushing motivation (12). A previous survey among Indian schoolchildren revealed that a majority preferred red-colored toothpaste with a fruity smell and a sweet taste, demonstrating how sensory customization can enhance compliance (12). Despite such evidence, data on children's sensory preferences for toothpaste and toothbrushes remain limited in the Saudi context, where oral health behaviours and caries prevalence patterns differ markedly from those in other regions.

In Saudi Arabia, various studies have investigated oral hygiene practices across different demographic groups and have reported notable differences associated with age, gender, parental education, and socioeconomic status (8, 11, 15). Findings suggest that approximately 88% of Saudi children begin consistent oral hygiene practices only after the age of seven, corresponding with a high prevalence of dental caries among both children and adolescents (8). Al-Ansari reported in their systematic review that 70% of preschool children in Saudi Arabia had caries (16). Another review reported a DMFT index of 3.34 among preschool children in Saudi Arabia (17). These results highlight an urgent need to explore factors that can motivate younger children to adopt regular brushing habits. To interpret the children's preferences, social cognitive theory (SCT) was used. SCT theory proposes that changes in behavior develop through personal, behavioral, and environmental influences. The SCT construct supports children's and parents' brushing preferences. Observational learning describes how children acquire brushing routines by modeling the behaviour of parents, siblings, and caregivers. Reinforcement explains how the pleasant sensory experience of brushing can increase the likelihood that the behavior will recur. Environmental influences include the visual and sensory aspects of oral-care products, which ultimately shape children's preferences for specific toothpaste and toothbrushes.

Understanding preferences and perceptions of toothbrushes and toothpastes can provide valuable insights for developing culturally and behaviourally appropriate oral health interventions. By aligning preventive strategies with children's sensory expectations and interests, dentists and parents can promote better adherence to oral hygiene routines. Therefore, the present study was designed to assess children's awareness, behavior, and preferences regarding the selection and use of toothbrushes and toothpastes. The study aims to provide evidence on the influence of factors such as color, flavour, and brand choice on children's oral hygiene behavior. The findings are expected to guide manufacturers, educators, and policymakers in designing more engaging preventive programs and oral care products that foster long-term, healthy habits among children in Saudi Arabia.

## Material and method

### Study design

A descriptive cross-sectional survey design was adopted to assess children's behavior and preferences regarding the selection and use of toothbrushes and toothpastes. This design was chosen as it allows data collection from a defined population at a single point in time, providing a snapshot of prevailing oral hygiene behaviours and influencing factors. The study was conducted in the Pediatric Dentistry Clinics of King Khalid University, College of Dentistry, over a period of five months (September 2023–January 2024).

### Study setting

**Figure 1:**
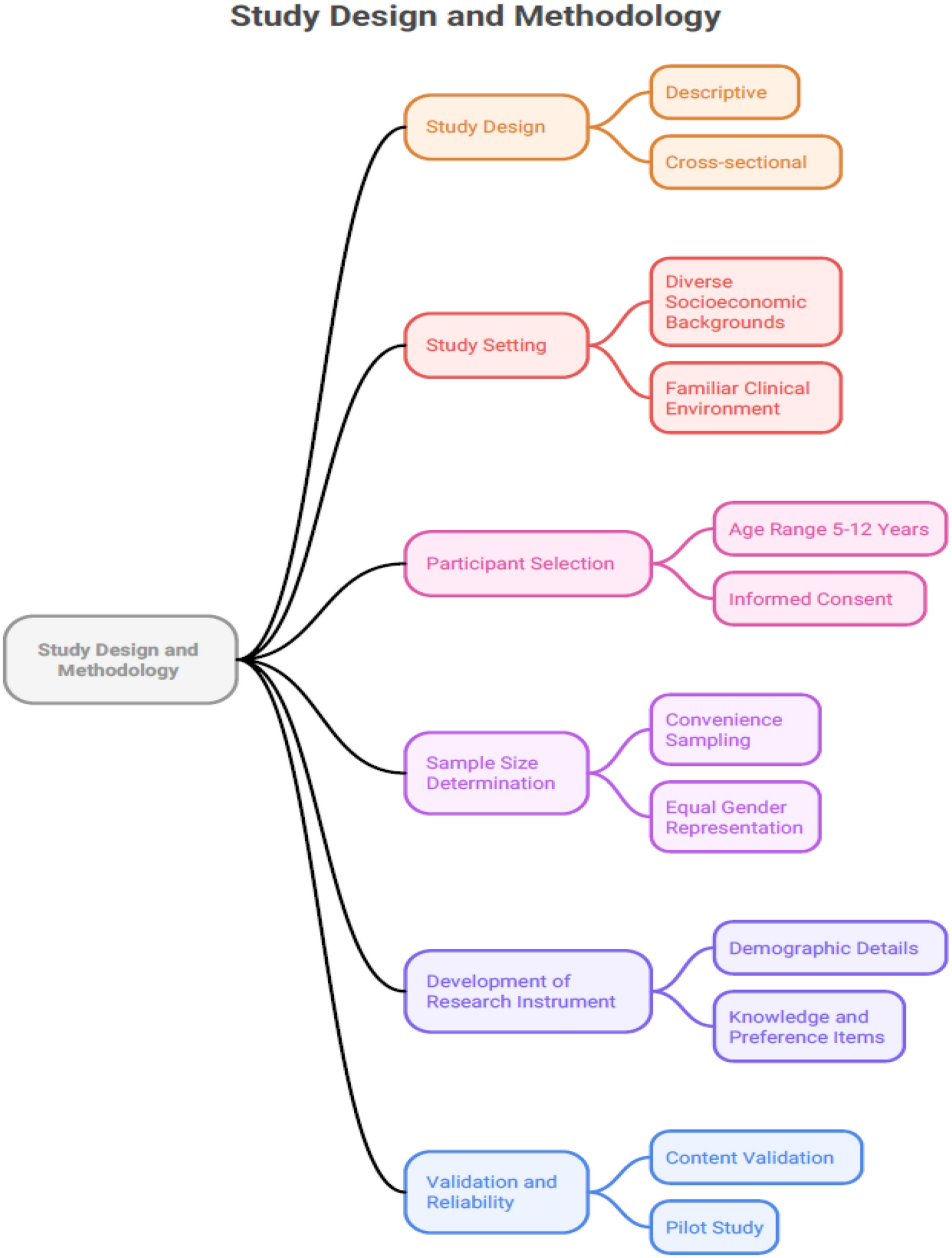
Study design and methodological approach of the current study (made with Napkin AI).

The Pediatric Dentistry Department serves a large number of children from different socioeconomic backgrounds, making it an ideal setting to assess oral hygiene awareness and preferences among the pediatric population. Data were collected in a familiar clinical environment, thereby reducing anxiety and enhancing response accuracy. Figure 1 illustrates the complete methodological pathway of the current cross-sectional study.

### Participant selection

Children aged 5–12 years visiting the Pediatric Dentistry Clinics during the study period were screened for eligibility. Inclusion criteria comprised: Children within the specified age range, Regular attendance at the Pediatric dental clinic for routine checkups or treatment, and Parents or legal guardians who were willing to provide informed written consent and assist in questionnaire completion when necessary. Exclusion criteria included: Children with physical or mental disabilities that could interfere with their ability to understand or respond appropriately to the questionnaire, Children or parents unwilling to participate or return incomplete questionnaires.

The selected age range was chosen because children aged 5–12 years are in the mixed dentition stage, when they begin developing independent brushing habits and product preferences, making them an appropriate group for behavioral assessment.

### Sample size determination

A total of 500 children were recruited through convenience sampling based on clinic attendance during the study period. Equal representation of both male and female participants was ensured to minimize gender bias. The study was conducted at King Khalid Hospital, one of the largest dental hospitals in the Southern Saudi region, where approximately 400 children attend as outpatients per week. The sample size was estimated to provide adequate power (80%) to detect statistically significant differences between categorical variables, assuming a 95% confidence interval and a 5% margin of error. This sample size also aligns with comparable cross-sectional oral health surveys in Pediatric populations.

### Development of the research instrument

A self-administered, structured questionnaire was developed in English and translated into the local language for better understanding among parents and children. The questionnaire comprised 17 items, divided into two main sections:

Demographic details – age, gender, and parental educational background.

Preference items – covering aspects of toothbrush and toothpaste use, including:

- Frequency and duration of toothbrushing.
- Type and brand of toothbrush and toothpaste used.
- Criteria for toothbrush selection (bristle type, handle design, color).
- Frequency and reason for toothbrush replacement.
- Preference for toothpaste flavour, color, and brand.
- Influence of advertisements, parents, or peers in product selection.

**Figure 2:**
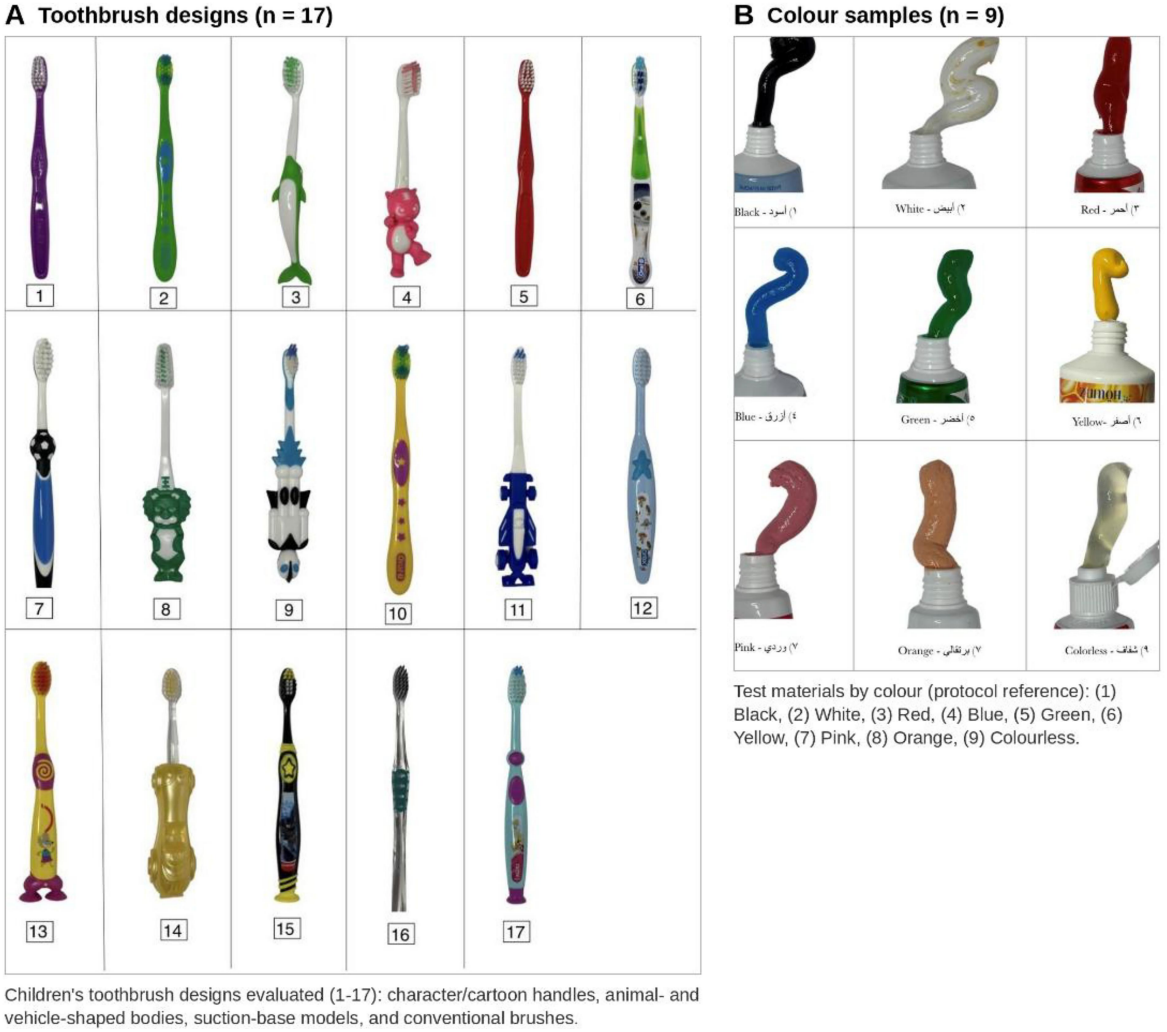
**(A)** Toothbrush designs for preference; **(B)** color preference given to children.

To enhance comprehension among younger participants, illustrative photographs of toothbrushes and toothpaste tubes were embedded within the questionnaire (Figure 2). This visual component helped minimize misinterpretation and increased children's engagement.

### Validation and reliability of the instrument

The draft questionnaire was subjected to content validation by a panel of five subject-matter experts in Pediatric Dentistry and Dental Public Health. Each item was evaluated for relevance, clarity, and simplicity. Modifications were made based on expert suggestions to improve readability and eliminate ambiguous items.

A pilot study was conducted on 30 children (not included in the final sample) aged 5–7 years to assess feasibility, comprehension, and completion time. Comprehension was assessed by asking children to restate items in their own words, using the embedded visual aids. Moreover, younger children received reading assistance from trained interns instructed not to influence answers, and, finally, we acknowledge that assisted responding may shift answers toward perceived-correct options. Based on feedback, minor revisions in language and format were incorporated. Internal consistency reliability was evaluated using Cronbach's alpha, yielding *α* = 0.85, indicating high internal consistency.

### Data collection procedure

Data collection was conducted in a dedicated area within the Pediatric Dentistry Clinic to ensure a comfortable, distraction-free environment. After explaining the study purpose, written informed consent was obtained from the parents or guardians. For younger children, the investigator or trained dental interns assisted in reading and explaining questions when required, without influencing their responses. Examiners also noted parental influence on children's answers. However, around 5% of preschoolers had asked their parents for answers. Each questionnaire required approximately 10–15 min to complete. Completed questionnaires were checked for completeness and consistency before data entry.

### Ethical considerations

Ethical clearance for the study was obtained from the Institutional Ethical Committee of King Khalid University, College of Dentistry (Approval No.: IRB/2025/ETH/2023-24/002). All procedures were conducted in accordance with the principles of the Declaration of Helsinki (2013 revision). Participation was entirely voluntary, and confidentiality was maintained throughout the study. Parents were informed about the study objectives, written informed consent was obtained, and anonymity was assured. Moreover, they were informed that they could withdraw their child from the study at any time without consequence.

### Data management and statistical analysis

All collected data were coded and entered into IBM SPSS Statistics for Windows, Version 20.0 (SPSS Inc., Chicago, IL, USA). Descriptive statistics such as frequencies and percentages were computed to summarize categorical variables. The Chi-square test was employed to determine associations between demographic factors (such as age and gender) and preference responses. A *p*-value of less than 0.05 was considered statistically significant. Data were cross-checked for accuracy and completeness prior to analysis to ensure data integrity.

## Results

Table 1 presents the age distribution of 500 children (250 males and 250 females) who participated in the study. The ages ranged from 5 to 12 years, with a mean age of 8.40 ± 2.22 years for females and 7.47 ± 1.48 years for males. The chi-square test value (1.115) and *p*-value (0.062) indicate that the difference in mean age between male and female participants was not statistically significant (*p* > 0.05), confirming that both groups were comparable in age.

**Table 1:** Distribution of study subjects according to age groups among both males and females.

Age (years)	Female *n* (%)	Male *n* (%)	Total *n* (%)
5	10 (4.0)	17 (6.8)	27 (5.4)
6	55 (22.0)	62 (24.8)	117 (23.4)
7	59 (23.6)	49 (19.6)	108 (21.6)
8	19 (7.6)	60 (24.0)	79 (15.8)
9	8 (3.2)	38 (15.2)	46 (9.2)
10	24 (9.6)	19 (7.6)	43 (8.6)
11	58 (23.2)	4 (1.6)	62 (12.4)
12	17 (6.8)	1 (0.4)	18 (3.6)
Total	250 (100.0)	250 (100.0)	500 (100.0)
χ^2^	1.165		
*P*-value	0.076*		

*p-value > 0.05 is insignificant.

The highest representation among females was in the 7-year age group (23.6%), followed by the 6-year (22.0%) and 11-year (23.2%) age groups. Among males, the 6-year (24.8%) and 8-year (24.0%) groups were the most prevalent. The Chi-square test result (1.165, *p* = 0.076) indicates that the age distributions of male and female participants were statistically similar.

Table 2 compares male and female responses regarding toothbrushing frequency, assistance, and reasons for brushing. A majority of participants in both groups reported brushing twice daily (54.0% females, 40.0% males), while 23.6% of females and 38.0% of males brushed once daily. The difference in brushing frequency was not significant (*p* = 0.051), though females showed slightly better brushing habits.

**Table 2:** Comparison among genders in relation to responses to the questions.

Response option	Female *n* (%)	Male *n* (%)	Total *n* (%)
**How often do you brush your teeth? (χ^2^ = 1.009, *p* = 0.051)**			
Once a day	59 (23.6)	95 (38.0)	154 (30.8)
Other	56 (22.4)	55 (22.0)	111 (22.2)
Twice a day	135 (54.0)	100 (40.0)	235 (47.0)
**Who helps you to brush your teeth? (χ^2^ = 2.178, *p* = 0.067)**			
I do it alone without assistance	228 (91.2)	220 (88.0)	448 (89.6)
My sibling	4 (1.6)	5 (2.0)	9 (1.8)
Nanny	1 (0.4)	2 (0.8)	3 (0.6)
Parent	17 (6.8)	23 (9.2)	40 (8.0)
**Why do you brush your teeth? (χ^2^ = 1.116, *p* = 0.079)**			
To clean your teeth	117 (46.8)	121 (48.4)	238 (47.6)
To feel freshness	18 (7.2)	19 (7.6)	37 (7.4)
To strengthen your teeth	49 (19.6)	51 (20.4)	100 (20.0)
To whiten your teeth	66 (26.4)	59 (23.6)	125 (25.0)
**Who selects the toothpaste? (χ^2^ = 1.661, *p* = 0.024)**			
I chose it by myself	92 (36.8)	116 (46.4)	208 (41.6)
My parents/guardian chose it	158 (63.2)	134 (53.6)	292 (58.4)
**How do you choose your toothpaste? (χ^2^ = 2.055, *p* = 0.079)**			
Colour	60 (24.0)	62 (24.8)	122 (24.4)
Flavour	77 (30.8)	85 (34.0)	162 (32.4)
Smell	67 (26.8)	72 (28.8)	139 (27.8)
Other	46 (18.4)	31 (12.4)	77 (15.4)
**Amount of toothpaste used?**			
Cover the entire bristles	53 (21.2)	48 (19.2)	101 (20.2)
Cover half of the bristles	93 (37.2)	84 (33.6)	177 (35.4)
In between bristles	47 (18.8)	60 (24.0)	107 (21.4)
Pea size	57 (22.8)	58 (23.2)	115 (23.0)
**Do you always use the same toothpaste? (χ^2^ = 1.667, *p* = 0.029)**			
No	77 (30.8)	63 (25.2)	140 (28.0)
Yes	173 (69.2)	187 (74.8)	360 (72.0)
**Reason for change (toothpaste)? (χ^2^ = 2.009, *p* = 0.018)**			
I didn't like the flavour	53 (21.2)	39 (15.6)	92 (18.4)
Others	197 (78.8)	211 (84.4)	408 (81.6)
**Favourite toothpaste colour? (χ^2^ = 1.514, *p* = 0.003)**			
Black	19 (7.6)	26 (10.4)	45 (9.0)
Blue	43 (17.2)	96 (38.4)	139 (27.8)
Colourless	15 (6.0)	7 (2.8)	22 (4.4)
Green	17 (6.8)	31 (12.4)	48 (9.6)
Orange	1 (0.4)	0 (0.0)	1 (0.2)
Pink	73 (29.2)	11 (4.4)	84 (16.8)
Red	40 (16.0)	37 (14.8)	77 (15.4)
White	30 (12.0)	22 (8.8)	52 (10.4)
Yellow	12 (4.8)	20 (8.0)	32 (6.4)
**Favourite flavour of toothpaste? (χ^2^ = 1.113, *p* = 0.052)**			
Grapes	23 (9.2)	24 (9.6)	47 (9.4)
Lemon	16 (6.4)	15 (6.0)	31 (6.2)
Mint	63 (25.2)	80 (32.0)	143 (28.6)
Orange	9 (3.6)	27 (10.8)	36 (7.2)
Strawberry	139 (55.2)	104 (41.6)	243 (48.6)
**Favourite toothpaste smell? (χ^2^ = 1.425, *p* = 0.053)**			
Grapes	54 (21.6)	51 (20.4)	105 (21.0)
Lemon	20 (8.0)	29 (11.6)	49 (9.8)
Mint	73 (29.2)	110 (44.0)	183 (36.6)
Orange	10 (4.0)	11 (4.4)	21 (4.2)
Strawberry	93 (37.2)	49 (19.6)	142 (28.4)
**What do you like about a toothbrush? (χ^2^ = 2.665, *p* = 0.047)**			
Colour	85 (34.0)	78 (31.2)	163 (32.6)
Shape and design of the toothbrush	78 (31.2)	87 (34.8)	165 (33.0)
My parents'/guardian's/sibling chose it	39 (15.6)	56 (22.4)	95 (19.0)
No preference	32 (12.8)	20 (8.0)	52 (10.4)
Other	16 (6.4)	9 (3.6)	25 (5.0)
Do you always use the same toothbrush? (χ^2^ = 2.282, *p* = see note*)			
No	84 (33.6)	79 (31.6)	163 (32.6)
Yes	166 (66.4)	171 (68.4)	337 (67.4)
**When do you change your toothbrush? (χ^2^ = 2.077, *p* = 0.054)**			
Every six months	44 (17.6)	52 (20.8)	96 (19.2)
Every three months	42 (16.8)	21 (8.4)	63 (12.6)
My parents/guardian changed it	66 (26.4)	75 (30.0)	141 (28.2)
Others	98 (39.2)	102 (40.8)	200 (40.0)
**Reason for changing toothbrush? (χ^2^ = 2.115, *p* = 0.036)**			
It became boring	43 (17.2)	59 (23.6)	102 (20.4)
The bristles become frayed	143 (57.2)	123 (49.2)	266 (53.2)
Others	64 (25.6)	68 (27.2)	132 (26.4)
**Favourite toothbrush colour? (χ^2^ = 1.324, *p* = 0.028)**			
Grey	1 (0.4)	2 (0.8)	3 (0.6)
Black	15 (6.0)	28 (11.2)	43 (8.6)
Blue	38 (15.2)	114 (45.6)	152 (30.4)
Green	10 (4.0)	37 (14.8)	47 (9.4)
Orange	3 (1.2)	2 (0.8)	5 (1.0)
Pink	95 (38.0)	2 (0.8)	97 (19.4)
Purple	54 (21.6)	2 (0.8)	56 (11.2)
Red	14 (5.6)	37 (14.8)	51 (10.2)
Transparent	5 (2.0)	2 (0.8)	7 (1.4)
White	8 (3.2)	8 (3.2)	16 (3.2)
Yellow	7 (2.8)	16 (6.4)	23 (4.6)
**What shape/design of toothbrush do you like? (χ^2^ = 1.526, *p* = 0.033)**			
Image 1	49 (19.6)	4 (1.6)	53 (10.6)
Image 2	7 (2.8)	12 (4.8)	19 (3.8)
Image 3	4 (1.6)	9 (3.6)	13 (2.6)
Image 4	110 (44.0)	9 (3.6)	119 (23.8)
Image 5	11 (4.4)	12 (4.8)	23 (4.6)
Image 6	1 (0.4)	2 (0.8)	3 (0.6)
Image 7	13 (5.2)	25 (10.0)	38 (7.6)
Image 8	1 (0.4)	10 (4.0)	11 (2.2)
Image 9	6 (2.4)	44 (17.6)	50 (10.0)
Image 10	3 (1.2)	2 (0.8)	5 (1.0)
Image 11	5 (2.0)	58 (23.2)	63 (12.6)
Image 12	5 (2.0)	8 (3.2)	13 (2.6)
Image 13	2 (0.8)	12 (4.8)	14 (2.8)
Image 14	4 (1.6)	4 (1.6)	8 (1.6)
Image 15	0 (0.0)	36 (14.4)	36 (7.2)
Image 16	15 (6.0)	3 (1.2)	18 (3.6)
Image 17	14 (5.6)	0 (0.0)	14 (2.8)

Values are *n* (%). Female and Male percentages are of each gender group (*n* = 250); Total percentages are of the full sample (*n* = 500). *t* and *p* values are the originally reported between-gender comparisons; *p < 0.05 was considered significant.

When asked who assisted them with brushing, most children brushed independently (91.2% of females, 88.0% of males). The difference was statistically insignificant (*p* = 0.067) (Table 2).

Regarding motivation for brushing, the primary reason was cleanliness (“to clean your teeth”) for nearly half of the participants, followed by whitening and strengthening. No significant gender variation was detected (*p* = 0.079) (Table 2).

A significantly higher proportion of males (46.4%) selected their toothpaste independently compared to females (36.8%), with *p* = 0.024. Most children (69.2% females, 74.8% males) reported consistently using the same toothpaste brand; this difference was also statistically significant (*p* = 0.029) (Table 2). The primary reason for changing toothpaste was dislike of the flavour, which was significant (*p* = 0.018).

The preferred toothpaste color differed significantly by gender (*p* = 0.003). Females favoured pink (29.2%), red (16.0%), and white (12.0%), while males preferred blue (38.4%) and green (12.4%) (Table 2).

When asked about favourite toothpaste Flavours, strawberry was most popular among females (55.2%), followed by mint (25.2%). Among males, mint (32.0%) and strawberry (41.6%) were dominant preferences. This variation approached significance (*p* = 0.052).

Similarly, in terms of toothpaste smell, females preferred strawberry (37.2%), while males preferred mint (44.0%), again showing a marginal difference (*p* = 0.053) (Table 2).

The color and shape/design of toothbrushes were the most influential selection factors, especially among females (*p* = 0.047) (Table 2). Approximately two-thirds of both groups used the same toothbrush regularly (*p* > 0.05), while frayed bristles (53.2%) were the most common reason for replacing a brush. Females, however, were more likely to replace their toothbrush because “it became boring” (*p* = 0.036).

Gender differences were significant in toothbrush color preference (*p* = 0.028). Females mainly preferred pink (38.0%) and purple (21.6%), while males preferred blue (45.6%), green (14.8%), and red (14.8%) (Table 2).

Regarding toothbrush design, females predominantly selected design type 4 (44.0%), whereas males showed varied preferences, including types 9 (17.6%), 11 (23.2%), and 15 (14.4%), yielding a statistically significant difference (*p* = 0.033) (Table 2).

**Figure 3:**
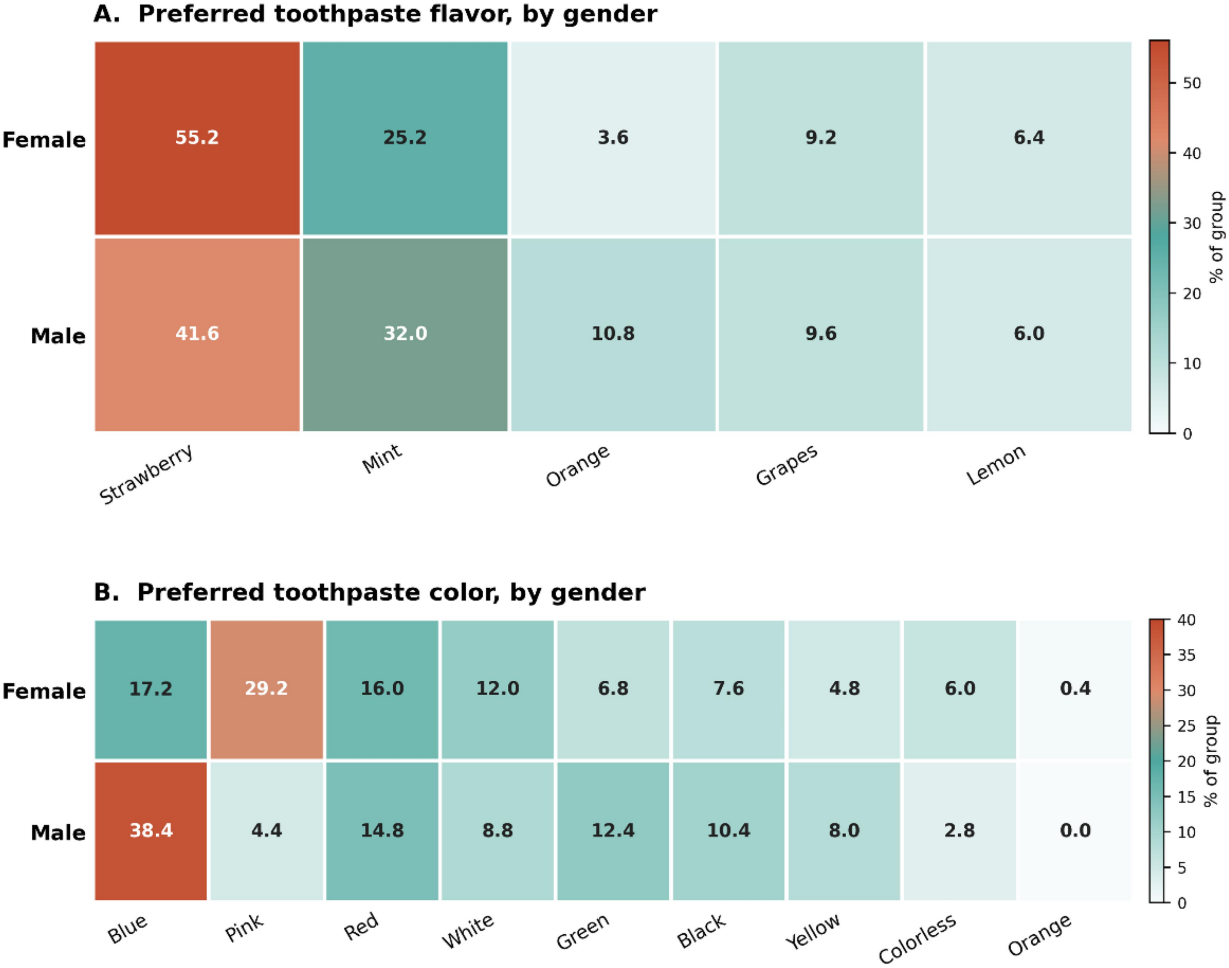
Sensory preference heat map: toothpaste color and flavor by gender. **(A)** Preffered toothpaste flavor, by gender. **(B)** Prefered toothpaste color by gender.

Interpreted through the environmental construct of SCT, the sensory-preference heat map (Figure 3) shows that toothpaste colour and flavour operated as gender-patterned stimuli, with 55.2% of female participants preferring strawberry-flavoured toothpaste and 29.2% preferring pink, whereas 32.0% of male participants preferred mint and 38.4% preferred blue. Within the SCT framework, these attributes represent the external cues hypothesized to attract children to oral-care products and reinforce repeated brushing.

## Discussion

Maintaining good oral and dental hygiene is a key determinant of general health and overall well-being. Effective plaque control through regular brushing and use of fluoridated toothpaste remains the cornerstone of preventing dental caries and periodontal diseases. The present study explored children's behaviour, preferences, and practices related to toothbrush and toothpaste use, providing insight into current trends in oral hygiene behaviour among Saudi children.

The findings revealed that most participants, regardless of gender, brushed their teeth twice daily without assistance, reflecting growing awareness and adherence to recommended oral hygiene practices. This improvement may result from the increasing number of oral health education programs in schools and communities, as well as from parents' and teachers' involvement in promoting preventive care. Similar to our findings, Matalon et al. reported that children often imitate their parents' oral hygiene habits, underscoring the strong parental influence on children's behavior (3). Trubey et al. further highlighted that parents act as role models in establishing brushing routines, underscoring the importance of parental guidance and reinforcement in achieving effective oral hygiene among children (18). However, a notable proportion of younger children in our study brushed without supervision, which could compromise proper cleaning and increase the risk of dental caries or excessive fluoride ingestion. Both the American Dental Association (ADA) and the American Academy of Pediatric Dentistry (AAPD) recommend parental supervision of brushing until at least eight years of age to ensure correct technique and appropriate toothpaste use (19).

The study also found that children's choice of toothbrush and toothpaste was strongly influenced by their parents. This dependence is positive, as informed parental decisions regarding toothbrush type, bristle texture, and timely replacement contribute to better oral health outcomes. The high proportion of children who reported brushing twice daily and appeared to follow parental routines is consistent with observational learning, in which children acquire brushing behavior through modeling by caregivers. Matalon et al. (3) and Trubey et al. (18) reported that children replicate their parents' oral hygiene habits. Unfortunately, earlier studies among Saudi populations have shown that inadequate parental awareness often leads to inappropriate toothbrush use and irregular replacement (11).

When asked about the amount of toothpaste used, around 20.2% of children reported using pea-sized toothpaste, while 40% used half of the toothbrush. A similar pattern was reported in a study of the Indian population, where awareness of the amount of toothpaste used was low (12). Educational programs should be conducted to raise awareness of excessive toothpaste use. Blinkhorn et al. supported the notion that, beyond proper toothbrush use, the quantity of toothpaste used daily is more important (20). Blinkhorn et al. observed mothers brushing their preschoolers and reported that only one-fourth of participants knew the amount of toothpaste used (20). Similar findings were reported in a survey of African American mothers, in which mothers were unaware of the amount of toothpaste to use (21). Policy makers and dental educators should consider these studies and should plan a preventive program focusing on the amount of toothpaste required for daily use (22).

As far as children are concerned, the role of color and flavor becomes prominent. When asked about their favorite toothpaste color, most of the boys preferred blue, while the girls preferred pink. The pronounced gender-patterned preferences for particular flavours and colours illustrate the role of the environmental construct of SCT. As sensory attributes function as salient stimuli that attract children to oral-care products and, by generating a pleasant brushing experience, act as positive reinforcement that increases the likelihood of repeated brushing. The finding that a significant number of children selected their own toothpaste and used a consistent brand further reflects the development of self-efficacy and outcome expectations, both central SCT constructs. Similarly, in a survey from India, 66% of girls preferred pink toothpaste, while 21% of boys preferred blue (12). However, in the study published among American children, the blue color was preferred among them (21). Children have limited cognitive capacity, which might influence their color preferences when selecting toothpaste. Moreover, children are sensitive to colour and always enjoy anything in their favourite colour. However, only a few studies have been published on the influence of color on children's psychology (23). Nevertheless, several studies find color as the advertising concept for selling a product (24, 25). Researchers in the current study used this concept to motivate children towards brushing. Hutching et al. noted that children generally prefer light colors. However, in the current study, children preferred blue and pink. Several studies have linked the favorite color to taste and demonstrated changes in decision-making (24, 25). In general, with particular regard to flavor, color brings a taste sensation like red: strong flavor; blue: mint; pink: sweet and sugary; yellow: spicy; orange: peppery. Hence, yellow and orange colors should not be used in toothpaste (12).

Flavour and colour emerged as dominant factors in toothpaste selection, with both male and female participants favouring strawberry-flavoured toothpaste. These findings are consistent with previous research showing that sensory attributes, flavour, color, and packaging encourage children to brush more frequently (12). While such preferences may enhance compliance, they also highlight the need for education emphasizing the importance of fluoridated toothpaste. The ADA and AAPD recommend using a smear-sized amount of fluoride toothpaste for children under three years and a pea-sized amount for those aged three to six years (22, 26, 27). Excessive toothpaste use may lead to fluoride ingestion and potential toxicity, reinforcing the importance of caregiver supervision and education.

Children in this study selected toothbrushes based on color and design, typically changing them only when their parents replaced them because the bristles had frayed. This indicates limited awareness of the recommended replacement interval of every three to four months. Therefore, dentists must educate both parents and children about selecting age-appropriate toothbrushes and replacing them regularly to ensure optimal plaque removal.

Overall, the present findings indicate encouraging levels of oral hygiene awareness among Saudi children, yet gaps persist in preferences, behaviour, and parental involvement. Strengthening school-based oral health promotion and community outreach programs can help consolidate these positive behavioural trends. Interactive educational tools, parental counseling, and dental professional involvement are crucial to maintaining long-term compliance with oral hygiene.

### Strengths and limitations

A major strength of this study is its large sample size (*n* = 500) and equal gender distribution, which enhances the reliability and representativeness of the findings. The use of a validated questionnaire (*α* = 0.85) ensured good internal consistency and dependable responses. Moreover, the inclusion of visual aids made the questionnaire age-appropriate and engaging for children.

However, the study's cross-sectional design limits its ability to establish causality between awareness and behavior. The self-reported nature of responses may have introduced social desirability bias, and the findings are limited to children visiting one university-based dental clinic, which may not reflect the entire Saudi Pediatric population. Future multicentre, longitudinal studies are recommended to assess the long-term impact of oral health education and behavioral interventions.

## Conclusion

The present study highlights a promising level of awareness and positive oral hygiene practices among Saudi children, with most brushing twice daily and demonstrating increasing autonomy in maintaining oral cleanliness. However, the findings also underscore the need for continued parental supervision, especially among younger children, to ensure proper brushing technique and appropriate use of fluoridated toothpaste. Children's preferences for toothbrush color, design, and toothpaste flavour, particularly fruity and sweet variants, play a key role in motivating regular brushing. Therefore, both educational initiatives and product development should take these sensory preferences into account to enhance compliance. Strengthening school-based oral health programs, along with active parental and professional involvement, can further improve oral hygiene behaviours. Ultimately, sustained education, awareness campaigns, and periodic reinforcement are essential to establish lifelong healthy oral habits and reduce the prevalence of preventable dental diseases among children.

## Data Availability

The raw data supporting the conclusions of this article will be made available by the authors, without undue reservation.
